# Fe-Nanoporous Carbon Derived from MIL-53(Fe): A Heterogeneous Catalyst for Mineralization of Organic Pollutants

**DOI:** 10.3390/nano9040641

**Published:** 2019-04-19

**Authors:** Thi Xuan Huong Le, Matthew G. Cowan, Martin Drobek, Mikhael Bechelany, Anne Julbe, Marc Cretin

**Affiliations:** 1 Institut Européen des Membranes, IEM UMR-5635 CNRS, ENSCM, University Montpellier, Place Eugène Bataillon, 34095 Montpellier CEDEX 5, France; quehuong2601@yahoo.com (T.X.H.L.); matthew.greig.cowan@gmail.com (M.G.C.); Martin.Drobek@univ-montp2.fr (M.D.); anne.julbe@umontpellier.fr (A.J.); marc.cretin@umontpellier.fr (M.C.); 2 Department of General Science, Van Lang University, 45 Nguyen Khac Nhu, District 1, Ho Chi Minh City 710200, Vietnam

**Keywords:** heterogeneous electro-Fenton process, metal-organic framework, dye removal, carbon, electrode

## Abstract

Catalytic electrodes were prepared via carbonization of MIL-53(Fe) on the surface of porous carbon felt electrodes (CF) for use in wastewater treatment by the heterogeneous electro-Fenton (EF) process. The best results were obtained when the carbon felt was pretreated with nitric acid, enhancing the affinity of the MIL-53(Fe) for the surface. Following a series of optimization experiments, carbonization conditions of 800 °C for 5 h were used to form Fe-nanoporous carbon (MOFs@CF). The as-prepared electrodes were used as both cathode and heterogeneous catalyst in the EF process for the mineralization of exemplar dye Acid Orange 7 (AO7). Total organic carbon (TOC) removal of 46.1% was obtained within 8 h of electrolysis at around neutral pH (6.5) and the electrode retained over 80% of its original efficiency over five treatment cycles.

## 1. Introduction

Advanced oxidation processes (AOPs) have been widely investigated for the removal of recalcitrant organic pollutants from wastewater [1,2]. Among them, the electro-Fenton (EF) process is considered as a promising technology for the treatment of contaminated industrial wastewater, a vital issue in both the developing and developed world [3]. Acid Orange 7 (AO7) is a typical example of azo dyes, characterized by the presence of the azo group (-N=N-), which are mainly used in the textile industry [4]. Azo dyes constitute the largest group of synthetic colorants used worldwide, making up around 70% of the mass of all dyestuffs [5]. When exposed into the environment via textile wastewater, they cause a threat to the aquatic medium because of their persistence and toxicity.

The EF process treats contaminated wastewater using classical Fenton’s reaction chemistry in which in situ hydroxyl radicals (OH) are produced via the Fenton’s reaction (Equation (1)) [6,7,8].

H_2_O_2_ + Fe^2+^ → Fe^3+^ + ^•^OH + OH^−^ (1)

Hydrogen peroxide (H_2_O_2_) is formed at the cathode by reduction of the dissolved oxygen (Equation (2)).

O_2_ + 2H^+^ + 2e^−^ → H_2_O_2_ (2)

After that, the organic pollutants (RH) are destroyed by the attack of ^•^OH radicals, according to Equation (3) [9].

^•^OH + RH → R + H_2_O (3)

In this process, Fe^2+^ ions can be regenerated electrochemically by Fe^3+^ reduction (Equation (4)):

Fe^3+^ + e^−^ → Fe^2+^ (4)

The application of EF technology to the treatment of AO7 in aqueous solutions was found to mineralize 92% of the dye [10]. The optimal applied current for AO7 decomposition was 300 mA, and the Fe^3+^ catalyst concentration was 0.1 mM in the solution. A mineralization reaction pathway of AO7 and solution toxicity during the treatment by EF degradation was also studied [10]. The mineralization led to the detoxification of treated AO7 solution at the end of the electrolysis step, proving the environmentally friendly ability of EF technology.

A remaining drawback to the application of EF technology is the requirement for relatively high concentrations of iron (0.1 to 0.2 mM) [11,12] to be added to the wastewater, which essentially results in exchanging one contaminant for another (i.e., replacing organic dyes with iron). In fact, applying a homogeneous EF process often results in the loss of soluble iron catalyst, thus requiring additional post-treatment operations prior to discharge. One approach to limiting the amount of iron released into solution is to use heterogeneous catalysts (instead of soluble iron salts) [13,14,15]. Previous strategies have used clay doped with iron sulfate or pyrite to treat a variety of contaminants. Similarly, heterogeneous catalysts such as iron alginate gel beads, core-shell nanoparticles [16], Fe_2_O_3_ modified kaolin, γ-FeOOH, and pyrrhotite were also investigated. These materials retain the iron on their surface or slowly release the iron into solution over time—prolonging the lifetime of the catalyst. Many of these experiments have been performed at strongly acidic pH (2 or 3), and it would be more convenient to perform water treatment at a pH closer to that of natural water. In addition, AOPs based on persulfates [17,18] have been used to treat refinery effluents under alkaline pH [19].

Another emerging iron source is provided by metal organic frameworks (MOFs). These materials have a periodic arrangement of metal nodes and the coordinating ligands can be chosen to affect the accessibility and oxidation level of iron atoms. Recent article catalogue the application of MOFs to the EF reaction [20]. These results show that MOFs have a good retention of performance over multiple (ca. 3–5) EF cycles and offer minimal leaching of iron into solutions. In most cases, the experiments are performed at pH 3–5, with only few studies exploring the pH values expected to be encountered during realistic operation with natural water (6.5–7.5) [20,21,22,23].

Of particular interest to our research group was solving the problem of particle agglomeration and investigating strategies to minimize iron leaching into the solution. We have recently explored methods to alter cheap carbon felt electrodes in order to optimize their performance in the EF process and avoid the introduction of particles to solution—avoiding any occurrence of particle agglomeration [24,25]. Immobilizing the catalyst on the carbon felt surface has little impact on performance, since most of the reaction occurs at the solid-liquid interface [26]. However, in a lab setting, the immobilized electrode does impact the reaction rate due to limited mass transport and long mixing times. Our research group has already explored the carbonization of carbon felt electrodes covered with CoFe Layered Double Hydroxides (LDH) [13]. The material provided good kinetics for the removal of AO7 from solution at pH 6.5, along with the ability to survive multiple cycles and achieve TOC removals of ca. 25% over 8 h. Furthermore, there was no appearance of iron oxide nanoparticles or agglomerates during the treatment. The improved results were attributed to a combination of: (i) large accessible pore volume, (ii) increased H_2_O_2_ production due to higher accessible surface area, and iii) surface catalyzed reactions—expanding the effective pH operating window.

In the present study, we explored the performance and stability of a carbon felt electrode modified with carbonized MIL-53(Fe), (MOF@CF). We explored the effect of varying thermal treatment temperature and MIL-53(Fe) loadings on the efficacy of the carbonized MOF@CF electrodes for removing AO7 from aqueous solution. We demonstrated that these materials can effectively mineralize AO7 around neutral pH (6.5) and retain over 80% of original efficiency over five treatment cycles. These results indicate that deriving modified electrodes from carbonized MOFs represents a promising approach, overcoming the disadvantages of homogeneous EF process, and thus contributing to the development of promising economical, efficient, and environmentally friendly technology in the area of biorefractory pollutant treatment.

## 2. Materials and Methods

### 2.1. Materials

Carbon felt was obtained from Alfa Aesar (A Johnson Matthey Company, 67300 Schiltigheim, France). The pretreated carbon felt was denoted as raw CF. AO7 (Orange II sodium salt), sodium sulfate (anhydrous, ≥99%), concentrated nitric acid, terephthalic acid (98%), iron (II) sulphate hepta-hydrate (99%), and DMF (99.8%) were purchased from Sigma Aldrich (38070 Saint-Quentin-Fallavier, France) and used without any further purification.

### 2.2. Equipment

The morphologies of the samples were characterized by scanning electron microscopy (SEM, Hitachi S-4800, Tokyo, Japan). X-ray diffraction (XRD) was used to assess the crystallinity of the prepared materials. In addition, other analysis methods such as X-ray photoelectron spectroscopy (XPS) (ESCALAB 250 Thermal Electron, Waltham, MA, USA) and energy dispersive X-ray spectroscopy (EDX), were applied to study the Fe-porous carbon. The N_2_ sorption-desorption isotherms were measured with Micromeritics ASAP 2010 equipment (Norcross, GA, USA, outgassing conditions: 200 °C-12 h).

### 2.3. Preparation of Carbonized MIL-53(Fe) Electrodes

The commercial CFs were cut with dimensions of 2.0 cm × 1.0 cm × 1.27 cm. They were cleaned either only in ethanol or pretreated first in concentrated nitric acid and then rinsed with deionized water before drying at 60 °C for 24 h. To grow MIL-53(Fe) on the CF surface, a mixture of FeCl_3_∙6H_2_O (2.155 g, 7.97 mmol) was dissolved in DMF (150 mL) with terephthalic acid (1.322 g, 7.96 mmol). The mixture was stirred until full dissolution was achieved. Aliquots of the solution (ca. 20–30 mL) were poured into Teflon-lined solvothermal pressure vessels. The pretreated CF was added, and the pressure vessels were sealed and then heated to 150 °C for 18 h. The treated CF was then removed from the pressure vessel and gently washed with methanol until the washing solution became clear. The treated CF was then dried in an oven at 105 °C for 2 h.

The modified carbon felt MIL-53(Fe)@CF was then carbonized in an oven under flowing N_2_ (200 mL min^−1^) at selected temperatures of 25 °C, 200 °C, 400 °C, 600 °C, 800 °C, or 1000 °C for a standard duration of 5 h. For a carbonization temperature of 800 °C, other durations have been investigated (15 min, 1 h, 5 h, 10 h), as reported in the discussion. The carbonized electrode materials were designated as pC@CFXXX/Yh where ‘XXX’ is the carbonization temperature and ‘Y’ its duration.

### 2.4. Mineralization of Azo Dye by Heterogeneous Electro-Fenton Process

The mineralization of AO7 dye by a heterogeneous EF process was carried out in a cylindrical glass cell with an effective volume of 200 mL. The cathode (pC@CF800/5h) and anode (Ti/Ni) were placed in parallel with a distance of 3 cm between them. The electrolysis solution was prepared by adding Na_2_SO_4_ (50 mM) into the AO7 solution (0.1 mM) and stirring vigorously until complete dilution of the supporting electrolyte. Compressed air (atmospheric air) was bubbled for 15 min at flow rate of 1 dm^3^ m^−1^ prior to the experiment in order to oxygenate the solution.

The mineralization of AO7 dye was started by applying the desired electrical current at −40 mA using DC power supply (Lambda Electronique, Melville, Long Island, NY, USA). The current remained constant during the electrolysis and aliquots for testing were withdrawn over the specific period of time. The AO7 mineralization was identified using a TOC-L CSH/CSN Shimadzu (Kyoto, Japan) analyzer to measure the total organic carbon (TOC) removal. Calibration curves for total carbon (TC) and inorganic carbon (IC) analysis were built up by automatic dilution of standard solutions of potassium hydrogenophtalate (TOC) and sodium hydrogen carbonate (IC). The mineralization was performed for 8 h, after which the electrolyte and dye solution was replaced with a freshly prepared solution.

## 3. Results and Discussion

The synthesis protocol adopted for the preparation of carbon felt electrodes modified by carbonized MOF material (pC@CF800/5h) involved two steps: (i) the growth of MIL-53(Fe) material on the surface of commercial carbon felts, and (ii) calcination (carbonization) of the MOF-modified carbon felts under a controlled atmosphere. The as-prepared electrode material was used in the heterogeneous EF process for the abatement of a model dye pollutant, acid Orange 7 (AO7). Hereafter, the key aspects related to the efficiency and stability of such a reaction system was discussed together with the characteristics of the electrodes. A special focus was placed on demonstrating that these materials can effectively mineralize AO7 around neutral pH (6.5), thus indicating that such electrode modification with carbonized MOFs represents a promising approach in the practical area of wastewater treatment.

### 3.1. Selection and Preparation of Electrode Materials

MIL-53(Fe) was selected for deposition onto the carbon felt electrode surface because carboxylate ligands provide an O6-coordination environment around the iron centers. Previous studies with MIL-100(Fe) have shown the effectiveness of carboxylate coordination environments for producing catalytic iron oxide sites [27,28,29].

**Figure 1:**
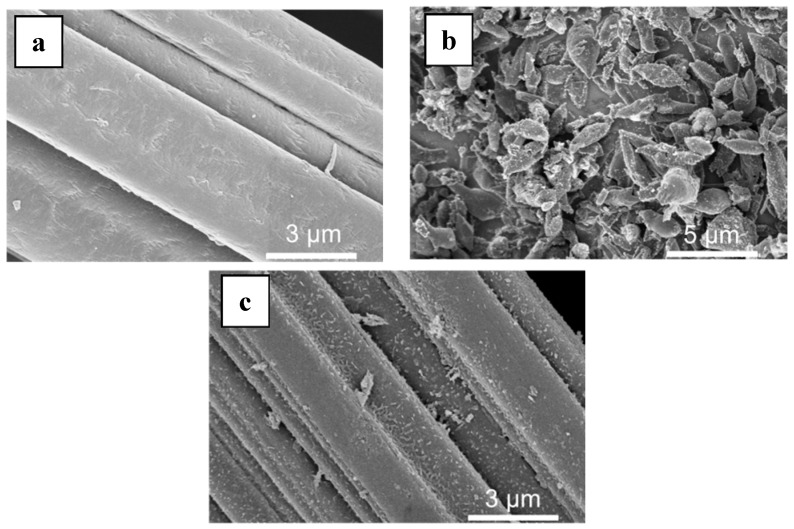
SEM of (**a**) raw carbon felt (CF), (**b**) CF covered with grown MIL-53(Fe) particles, (**c**) final Fe-MOFs@CF800/5h electrode.

MIL-53(Fe) was grown on the surface of a commercial carbon felt (CF) using a solvothermal synthesis protocol [30,31] in the presence of the carbon felt (Figure 1a) immersed in the reaction mixture. The synthesis led to the formation of MOF-based composite material with MIL-53(Fe) elongated crystals (2–3 µm in size) uniformly covering the carbon fibers (Figure 1b).

Initial experiments using commercial CF either without any pretreatment, or washed with ethanol, resulted in low loading of MIL-53(Fe) due to weak adhesion of the latter to the surface. To overcome this issue, we determined that pre-treatment with concentrated nitric acid led to improved attachment and increased retention of MIL-53(Fe). The as-treated electrode was washed with methanol until the washing solution was clear, ensuring that no loose material remained and could leach during the EF reaction. Using this method, MIL-53(Fe) loadings of up to 12 wt. % were achieved, producing modified carbon felts (MOF@CF).

**Figure 2:**
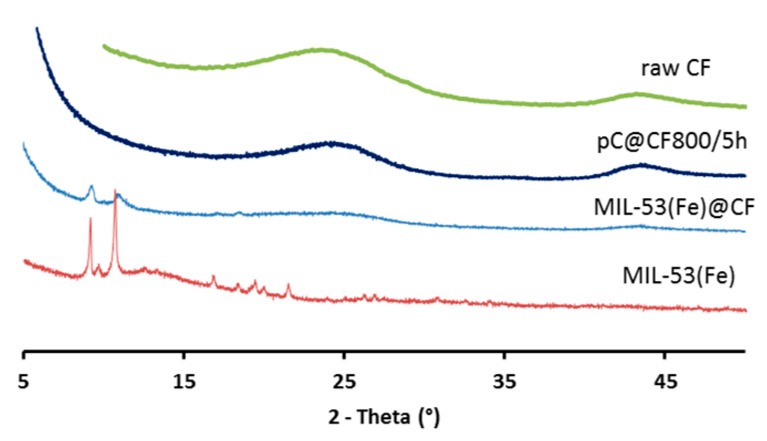
XRD patterns of raw CF, pC@CF800/5h electrode, virgin MIL-53(Fe)@CF sample and MIL-53(Fe) powder.

The formation and presence of MOFs was confirmed (Figure 2) by the characteristic XRD peaks (2*θ* = 9.26°, 11.1°, 16.38°, 17.2°) of MIL-53(Fe). Hence, under the selected reaction conditions, the synthesis resulted in MIL-53(Fe)@CF composite electrode material.

### 3.2. Effect of Carbonization Conditions on Electrode Performance

**Figure 3:**
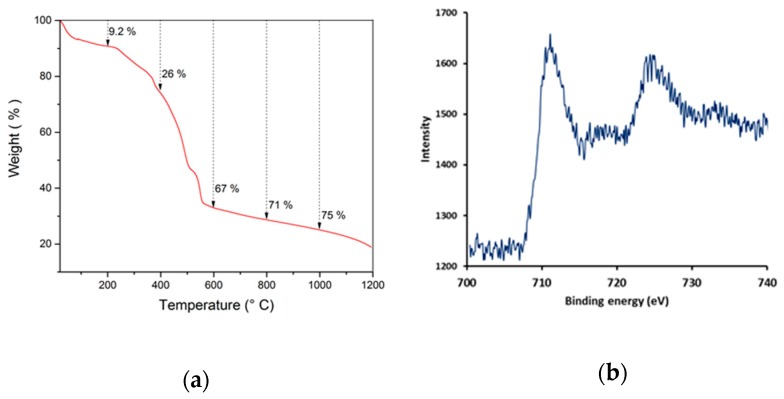
(**a**) TGA of MIL-53(Fe) powder under nitrogen with a heating rate of 5 °C/min, (**b**) XPS spectrum of pC@CF800/5h electrode.

The MIL-53(Fe)@CF samples were carbonized under a nitrogen atmosphere, in order to generate the catalytically active iron-doped porous carbon (pC) thin layers covering the surface of commercial CFs (Figure 1c). To define the optimal thermal treatment, thermogravimetric analysis (TGA) was used to determine the carbonization behavior of MIL-53(Fe)@CF under nitrogen versus temperature (Figure 3a).

As a result of this investigation, and as already detailed in the experimental section, we prepared a series of pC@CFXXX/5h samples by heating MIL-53(Fe)@CF under N_2_ at 25 °C, 200 °C, 400 °C, 600 °C, 800 °C, or 1000 °C for 5 h [32,33,34].

The performance of the pC@CFXXX/5h electrodes was evaluated by measuring the removal of total organic carbon from the system over an 8 h period (Table 1). The results indicate that carbonization at temperatures lower than 600 °C actually leads to more dissolved carbon entering the solution. We attribute this result to the dissolution of the remaining MIL-53(Fe) framework that was not fully carbonized under the selected conditions, as already observed in our studies where MFI zeolite was physically deposited onto an electrode surface [24]. However, above 600 °C the framework was successfully carbonized into iron oxide and carbon. We see a ca. 40% reduction in the TOC of the solution—indicative of the electro-Fenton process operating effectively with limited or no dissolution of the residual MIL-53(Fe). This observation suggests that the carbonized form of the MOF is relatively insoluble and/or more strongly adhered to the carbon felt.

Having established the optimal carbonization temperature, we performed carbonization at 800 °C for a duration varying between 15 min and 10 h. The results showed that positive TOC removal was obtained with pC@CF800/15 min, and the highest TOC removal (46%) was obtained with the carbon felt carbonized for 5 h. No additional benefit was observed after 10 h carbonization time.

XRD analysis (Figure 2) showed that crystallinity was removed in the pC@CF material (pC@CF800/5h), thus confirming the carbonization of MIL-53(Fe). The XPS analysis of the carbonized sample (Figure 3b) evidenced iron preservation, thus confirming the formation of the heterogenized Fe-based catalyst on the carbon felt surface. The binding energy at 725 eV corresponds to Fe2p1/2, while the peak at 711 eV is assigned to Fe2p2/3, thus confirming the presence of Fe_2_O_3_.

The virgin and carbonized CF electrodes were also characterized using N_2_ physisorption. The carbonized electrode had a surface area about 55 times higher than virgin carbon felt (S_BET_ = 0.1 vs. 5.5 m^2^/g) forming a porous structure consisting mainly of micropores (pore volume = 0.012 cm^2^/g).

### 3.3. Effect of MIL-53(Fe) Concentration on Electrode Performance

**Figure 4:**
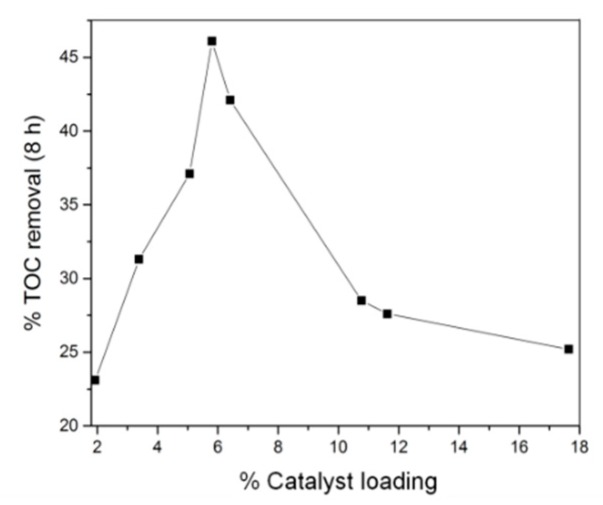
Percent total organic carbon (TOC) removal of 200 mL Acid Orange 7 (AO7) (0.1 mM) after 8 h treatment by heterogeneous EF process as a function of catalyst loading deposited on carbonized CF@MIL-53(Fe) cathode.

Varying the reaction time of solvothermal synthesis allowed some degree of control over the final density of MIL-53(Fe) on the carbon felt surface. After subsequent carbonization, this translates to a control over the density of iron oxide in the modified carbon felt electrode. Using this strategy, we observed that an increase of the catalyst amount increases electrode performance until a loading of ca. 5.8%, which allows TOC removal of ~46% to be achieved (Figure 4) within 8 h of electrolysis. Increasing the loading past this point caused a reduction in TOC removal. This result can be explained by considering that increasing the concentration of iron sites leads to the competing reaction of electrochemically generated iron(II) with hydroxyl radicals to produce hydroxide ions incapable of further destroying the organic material [35].

### 3.4. Stability of the Carbonized MIL-53(Fe) Electrode

**Figure 5:**
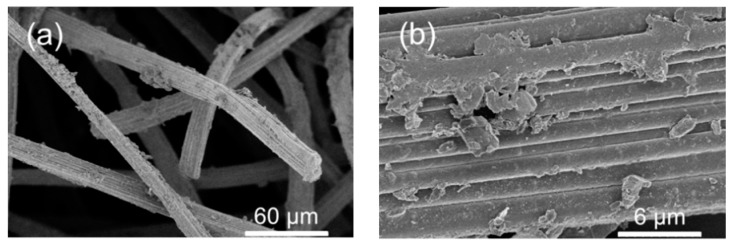
(**a**,**b**) SEM observations of pC@CF800/5h cathode after ten cycles heterogeneous EF process.

The stability of the carbonized MIL-53(Fe) electrode (pC@CF800/5h) was investigated by measuring the leaching of iron into solution and the retention of performance in TOC removal. The conditions were reasonably harsh, with each experiment involving exposure to a vigorously stirred solution at pH 6.5 for 8 h. In such conditions, the electrodes produced from MIL-53(Fe) grown directly onto the electrode surface retained TOC removal of up to 29% after 10 cycles (Table 2). Moreover, the TOC removal was almost double that of electrode materials prepared from physically deposited MFI zeolite (27% after 8 h, 1 cycle) as published recently by our research group [24]. The partial decline of the catalytic activity could be explained by a washing of the iron catalyst out of the electrode during its repetitive use in several EF cycles (Table 3) and/or electrode fouling (Figure 5) limiting the access to the catalytic sites. Hence, from an industrial point of view the stability of the electrode is still relatively too low for possible larger scale applications.

Finally, the specific energy consumption per unit TOC mass (EC_TOC_) was also evaluated according to Equation (5) [36]:

(5) $${\mathrm{EC}}_{\mathrm{TOC}}\;(\mathrm{kWh}\;g^{-1}\;\mathrm{TOC})=\frac{\mathrm{VIt}}{{\left(\Delta \mathrm{TOC}\right)}_{\exp}V_{s}}$$

where V is the average cell voltage (V), I is the applied current (A), t is the electrolysis time (h), and Vs is the solution volume (L).

At ~46% TOC removal, the EC_TOC_ was 0.605 kWh g^−1^, hence comparable value to that already observed for another heterogeneous EF process using an Fe-alginate beads catalyst (0.87 kWh·g^−1^ EC_TOC_ at 60% TOC removal) [37].

To conclude on the efficiency of the developed heterogeneous EF system, comparisons with other data available in the literature with similar Fe-based catalytic sources such as Fe@Fe_2_O_3_, pyrite, CoFe/Fe^II^Fe^III^-LDH etc. are summarized in Table 4. It must be underlined that compared to other studies in the area of EF processes applying high electric current density or other types of anode (e.g., boron-doped diamond (BDD) anode), the present work offers attractive results of ~46% TOC removal (AO7, 0.1 mM) at low current density of 3.8 mA cm^−2^ and nearly neutral pH (6.5); thus, proving the system to be very promising for the removal of organic pollutants from the natural water environment. Further improvements of the system (e.g., higher catalyst loading, application of other MOF materials, different deposition protocols, carbonization treatments etc.) leading to more stable and active heterogenized catalysts will be the subject of our future communication. It will focus on improving the system’s performance and reducing the energy consumption for total degradation of water pollutants.

## 4. Conclusions

We have presented the growth of a metal organic framework (MIL-53(Fe)) on the surface of a carbon felt electrode, followed by its carbonization in order to produce a recyclable catalytic electrode for the electro-Fenton reaction. We have systematically investigated preparation conditions, establishing that catalyst loadings, thermolysis temperature, and thermolysis time play an important role in the efficiency of the final material. Electrodes prepared under the optimal conditions of 5.8% catalyst loading with carbonization at 800 °C for 5 h achieved TOC removal of ca. 45% under the experimental conditions with the model electrolyte. Our focus is now on both exploring the wide range of MOFs available for improving the efficiency of the catalytic site and investigating system performance under realistic operating conditions.

## Figures and Tables

**Table 1:** Effect of carbonization temperature and duration on the electro-Fenton performance of carbon felt electrodes modified with carbonized MIL-53(Fe).

	Temperature (5 h Heating)						Time (at 800 °C)			
	25 °C	200 °C	400 °C	600 °C	800 °C	1000 °C	15 min	1 h	5 h	10 h
% TOC removal	−94.4	−75.5	−63.8	42.5	46.1	46.3	19.9	36.3	46.1	44.6

**Table 2:** Amount of Fe leaching in solution and percent TOC removal after 8 h heterogeneous electro-Fenton (EF) treatment using pC@CF800/5h cathode.

	Cycle 1	Cycle 3	Cycle 5	Cycle 10
[Fe] (mg L^−1^)	1.177	1.076	0.810	0.531
% TOC removal	46.1	43.3	37.5	29.1

**Table 3:** Elemental composition of raw CF, pC@CF800/5h electrode after 1 cycle and pC@CF800/5h electrode after 10 cycles heterogeneous EF process.

Element (% mass)	Raw CF	pC@CF800/5h after 1 Cycle	pC@CF800/5h after 10 Cycles
C	96	82	86
O	4	8	7
Fe	-	10	4
other elements	-	-	3

**Table 4:** Summary of catats for heterogeneous EF process using CF cathode.

Catalyst	Experimental Conditions		% Removal of Organic Pollutants	Ref.
	Applied Current or Potential	Electrolysis Time		
Fe@Fe_2_O_3_	Power density value (p) = 4.35 W m^−2^	10 h	81% of 17β-estradiol and 56% of 17α-ethynyl-estradiol (20 µg L^−1^)	[38]
Pyrite	I = 300 mA	6 h	90% TOC of tyrosol (0.30 mM)	[39]
Fe_2_O_3_-KLN	I = 60 mA	40 min	80% of enoxacin (0.25 mM)	[40]
*γ*-Fe_2_O_3_/Fe_3_O_4_	E = 2 V	600 min	85% removal of diclofenac and 36% TOC (0.62 mg L^−1^)	[41]
Pyrite	I = 300 mA	8 h	~100% TOC of 4-amino-3-hydroxy-2-p-tolylazo-naphthalene-1-sulfonic acid (175 mg L^−1^)	[42]
Fe@Fe_2_O_3_	13.8–25.9 kC	12 h	95% Rhodamine B (15 mg L^−^^1^) and 90% TOC	[43]
Fe@Fe_2_O_3_	E = 600 mV	48 h	78.05% COD removal of medicinal herbs wastewater	[44]
[CoFe-LDH	i = 4.2 mA cm^−2^	8 h	66% TOC of Acid Orange 7 (0.1 mM, pH = 7.1)	[13]
Chalcopyrite	I = 300 mA	8 h	98% TOC of antibiotic tetracycline (0.20 mM)	[45]
Fe^II^Fe^III^-LDH	i = 7.5 mA cm^−2^	8 h	93% TOC of sulfamethoxazole (0.2 mM, pH = 6)	[14]
Fe-MFI zeolite	i = 3.8 mA cm^−2^	8 h	26.6% TOC of Acid Orange 7 (0.1 mM, pH = 6.5)	[24]
pC(Fe)	i = 3.8 mA cm^−2^	8 h	46.1% TOC of Acid Orange 7 (0.1 mM, pH = 6.5)	*Present study*
